# Autofluorescence spectroscopy in redox monitoring across cell confluencies

**DOI:** 10.1371/journal.pone.0226757

**Published:** 2019-12-18

**Authors:** Derrick Yong, Ahmad Amirul Abdul Rahim, Chaw Su Thwin, Sixun Chen, Weichao Zhai, May Win Naing

**Affiliations:** Bio-Manufacturing Group, Singapore Institute of Manufacturing Technology, Singapore, Singapore; Institute of Human Virology, UNITED STATES

## Abstract

Patient-specific therapies require that cells be manufactured in multiple batches of small volumes, making it a challenge for conventional modes of quality control. The added complexity of inherent variability (even within batches) necessitates constant monitoring to ensure comparable end products. Hence, it is critical that new non-destructive modalities of cell monitoring be developed. Here, we study, for the first time, the use of optical spectroscopy in the determination of cellular redox across cell confluencies by exploiting the autofluorescence properties of molecules found natively within cells. This was achieved through a simple retrofitting of a standard inverted fluorescence microscope with a spectrometer output and an appropriate fluorescence filter cube. Through spectral decomposition on the acquired autofluorescence spectra, we are able to further discern the relative contributions of the different molecules, namely flavin adenine dinucleotide (FAD) and reduced nicotinamide adenine dinucleotide (NADH). This is then quantifiable as redox ratios (RR) that represent the extent of oxidation to reduction based upon the optically measured quantities of FAD and NADH. Results show that RR decreases with increasing cell confluency, which we attribute to several inter-related cellular processes. We validated the relationship between RR, metabolism and cell confluency through bio-chemical and viability assays. Live-dead and DNA damage studies were further conducted to substantiate that our measurement process had negligible effects on the cells. In this study, we demonstrate that autofluorescence spectroscopy-derived RR can serve as a rapid, non-destructive and label-free surrogate to cell metabolism measurements. This was further used to establish a relationship between cell metabolism and cellular redox across cell confluencies, and could potentially be employed as an indicator of quality in cell therapy manufacturing.

## Introduction

The cell therapy industry has garnered significant momentum in recent years, pivoting on the promise that cell-based therapies hold in treating conditions where conventional approaches have failed [1]. As therapies make the leap from lab to bedside, a major challenge highlighted in the manufacturing of such therapies lies with establishing quality and developing control processes [2, 3]. With patient-specific therapies, there is added complexity as a result of the inherent variability of cells (donor-to-donor variation) [4]. The current standards of using destructive testing is time-consuming, costly and essentially reduces the available dosage for the patient. The development or adoption of monitoring tools in such a context is well aligned with FDA’s guidelines under the Process Analytical Technology (PAT) framework [5]. Ideally, monitoring methods to ensure quality of such products should be achievable *in situ*, non-destructively and in real-time [6]. Optical spectroscopy is a tool that meets these requirements [7, 8], with effects like Raman scattering [9] and autofluorescence [10] offering specificity under a label-free modality.

Cells contain bio-molecules capable of emitting fluorescence; This is known as cellular autofluorescence [11]. These cell-endogenous fluorophores are the very same bio-molecules responsible for a host of cellular processes that govern cell functions and metabolic activities. Different fluorophores can be differentiated by their spectral distribution of emissions, with the amount of emission further corresponding to their respective quantities. Since the pioneering work by Chance *et al*. [12], where a relationship was established between cellular autofluorescence and cellular metabolic processes, cell-endogenous fluorophores have been successfully used as biomarkers in the non-destructive and real-time determination of cell characteristics. Numerous adoptions have thus been made in biomedical research and diagnosis [13], with notable applications in the identification of stem cell differentiation [14, 15] as well as the detection of diseases such as cancer [16, 17] and Alzheimer’s [18]. These applications have been enabled by optical techniques such as multi-photon microscopy cum spectroscopy [15, 19] and fluorescence lifetime imaging microscopy [20, 21]. Aside from these capital- and skill-intensive techniques, which offer in depth details more pertinent to the fundamental understanding of the biosciences, more broadly adoptable and economical methods like multispectral microscopy [22] and autofluorescence spectroscopy [10, 23] have also been reported as practical alternatives.

One measurand of interest is cellular redox [10, 19, 24, 25], which offers a direct indication of the cells’ metabolic activity and redox state, potentially serving as a quality attribute for monitoring cells. This is quantified using a redox ratio (RR) that indicates the extent of oxidation against reduction based upon the optically measured amounts of metabolic co-enzymes—flavin adenine dinucleotide (FAD) and reduced nicotinamide adenine dinucleotide (NADH), correspondingly. In the context of cell manufacturing, probing for RR during the manufacturing process would allow the operator to regularly and quickly discern the metabolic and redox state of cells. This would allow for more quantitative and objective judgements to be made for optimization of output and establishing quality control. In contrast, the current primary method of non-destructively assessing cell quality, through cell morphology and numbers (or confluency for adherent cells), is very subjective.

In this work, we studied the use of autofluorescence spectroscopy as a non-destructive and label-free method of determining cell metabolic activity and redox state. We acquired RR from cells at different confluencies, examined the relationship between RR and various cell attributes, and assessed their applicability as quality attributes for monitoring. Lastly, we validated our measurements against conventional bio-chemical assays and ascertained that our method did not adversely affect cells.

## Methods and materials

### Cell culture

WS1 human skin fibroblast cells (CRL1502, ATCC, USA) at Passage 4 were used. Cells were cultured with complete culture media comprising 90% Minimum Essential Medium Eagle (PAN-Biotech, Germany) with Earle’s Balanced Salt Solution, L-Glutamine, Sodium Pyruvate, Sodium Bicarbonate and Non-Essential Amino Acids as well as 10% Fetal Bovine Serum (HyClone, Life Technologies, USA). Cultures were maintained within 75cm^2^ cell culture flasks (Corning, USA) in a CO_2_ incubator (Forma Steri-Cycle i160, Thermo Fisher Scientific, USA) set at 37°C, 95% humidity and 5% CO_2_, with its culture media changed every other day. Cells were cultured to 90% confluency before washing with Phosphate Buffered Saline (PBS) (17-516F, Lonza, USA) and harvesting with Trypsin EDTA Solution B (0.25%), EDTA (0.05%) (Biological Industries, USA). Cell concentration was determined with an automated cell counter (EVE Automated Cell Counter, NanoEnTek, South Korea). Cell suspensions were mixed with an equal volume of Trypan Blue and transferred into cell counting slides (EVE Chamber Slides, NanoEnTek, South Korea) before being inserted into the automated cell counter. Cell suspensions were subsequently diluted to the required concentrations prior to seeding for microspectroscopy and the bio-chemical assays. All cell culture work and related procedures were performed within a Biosafety Cabinet (BSC) (1300 Series A2, Thermo Fisher Scientific, USA).

### Autofluorescence microspectroscopy

**Fig 1:**
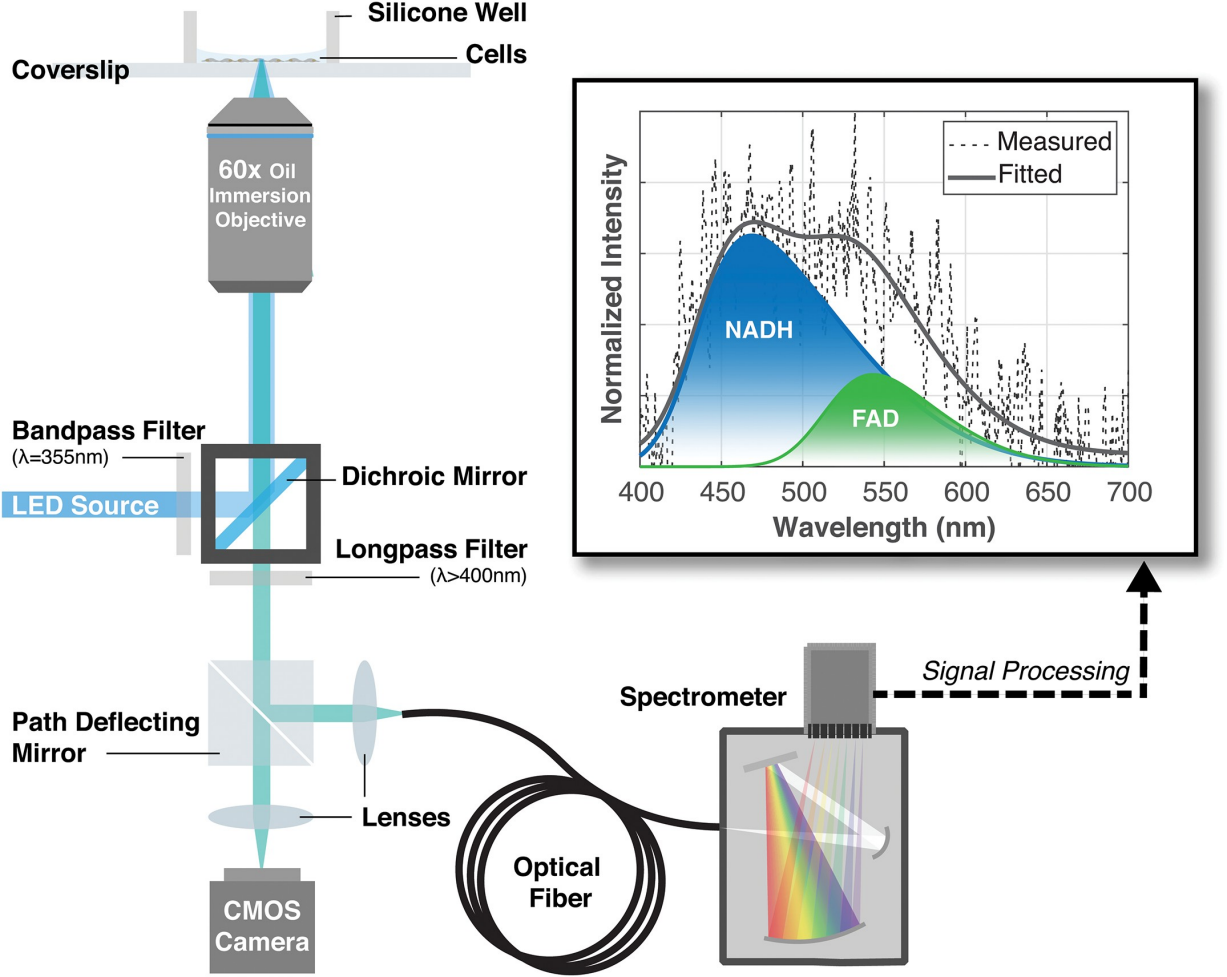
Schematics of microspectroscopy setup. Setup comprises an inverted fluorescence microscope with a custom fluorescence filter cube and retrofitted with an output port that is fiber-coupled to a spectrometer. Cells adhered to glass coverslips are immersed in imaging solution during measurements. Inset: Autofluorescence spectra collected from live cells. The measured and fitted spectra are represented by a black-dotted and a solid-grey curve, correspondingly. The constituent emissions of NADH and FAD are indicated by blue and green curves respectively.

Microspectroscopy was performed using an inverted fluorescence microscope (IX73, Olympus, Japan) after a simple retrofitting. Schematics are shown in Fig 1. Excitation was supplied by a LED illumination source (wLS, QImaging, Canada) with a center wavelength of 365nm. In order to excite and collect cellular autofluorescence, a customized fluorescence filter cube was assembled with optics obtained from Thorlabs (USA). This comprised an excitation bandpass filter with a center wavelength of 355nm and FWHM of 10nm (FLH355-10); an emission longpass filter with a cut-on wavelength of 400nm (FELH0400); and a dichroic mirror that reflects wavelengths below 407nm and transmits wavelengths above 425nm (MD416). Spectral measurements were obtained with a spectrometer—comprising a spectrograph (Shamrock SR-303i, Andor, UK) and spectroscopy CCD (Newton 920, Andor, UK)—that was fiber-coupled to the microscope’s camera port via a fiber collimator (F280SMA-A, Thorlabs, USA).

For autofluorescence microspectroscopy, cells between Passage 4 to 6 were seeded on 60mm×24mm 0.175mm-thick glass coverslips (D263M, Schott, Germany) within square silicone wells with growth areas of ∼1.9cm^2^ and heights of 13.8mm. Silicone wells were fabricated from medical grade silicone (Silpuran 6000/10, Wacker Chemie AG, Germany). Before each use, silicone wells were sterilized by autoclaving at 121°C for 20min and air-dried in a BSC for 1h. Silicone wells were then set on the glass coverslips before undergoing UV sterilization in the BSC for 1h. (These were filled with culture media and observed under the microscope for a week to validate the effectiveness of the preparation and sterilization process.) Prior to seeding, the set silicone wells were washed with 1ml of PBS and incubated with 1ml of culture media in the CO_2_ incubator. Cells were seeded at a concentration of 3.0×10^4^cells/ml with 1ml of cell suspension transferred to each coverslip within the confines of the silicone wells. These were then incubated in the CO_2_ incubator with their culture media changed every other day until extracted for autofluorescence microspectroscopy. Coverslips were taken out of the incubator for microspectroscopy after varying durations of incubation, ranging from 15h to 180h (∼7 days). The different durations of incubation resulted in cultures with different cell confluencies. Prior to microspectroscopy, culture media in silicone wells was removed and the cells were washed twice with 1ml of PBS with Ca^2+^ and Mg^2+^ (17-513F, Lonza, Belgium). 100*μ*l of live cell imaging solution (A14291DJ, Thermo Fisher Scientific, USA) was then added. Coverslips were then mounted on the stage of the fluorescence microscope for microspectroscopy. Prior to microspectroscopy, five phase contrast images were obtained per coverslips at random locations through a 4× objective. Cell confluency was subseqeuently determined via visual inspection of these images by at least two individuals. All other mentions of cell confluency were determined in a similar fashion.

Autofluorescence spectra were obtained by first locating the region of interest on the coverslip through the microscope’s camera before deflecting the collected light to the spectrometer. Each spectral measurement was acquired via the spectrometer over the integration time of 2s with a 100*μ*m slit size. This was done through a 60× oil-immersion super apochromat objective (UPLSAPO60XO, Olympus, Japan) together with a low autofluorescence immersion oil (IMMOIL-F30CC, Olympus, Japan). To minimize the exposure of cells to the excitation source, we first selected the region of interest under low bright field illumination before switching to the LED illumination for image capture and spectral measurements. For each sample, at least ten measurements (N≥10) were made at different locations on the cell-covered regions of the coverslip. This was followed by background measurements of a 100*μ*l drop of imaging solution at a clean region of the same coverslip. Background measurements were repeated five times per coverslip. Measurements were made over four biological repeats, with each repeat comprising between 8 to 16 samples to obtain at least three sets of data per reported confluency.

### Spectral decomposition and optical redox ratio

Collected autofluorescence spectra were processed using a MATLAB-based software developed in our laboratory. The software is designed to perform signal processing, background correction and spectral decomposition that breaks down a spectrum into its constituent emission spectra. The latter was based on a non-linear curve-fitting procedure by Croce *et al*. [10, 23]. In this work, we limited the spectral decomposition to just two cell-endogenous fluorophores of interest—reduced nicotinamide adenine dinucleotide (NADH) and flavin adenine dinucleotide (FAD).

Our software was trained to recognize the emissions of NADH and FAD using reference solutions. Varying concentrations of reference solutions were prepared by dissolving NADH (N8129, Sigma-Aldrich, USA) in Tris buffer at pH 8.0 (BUF-1414-500ml-pH8.0, 1st Base, Singapore) and FAD (F6625, Sigma-Aldrich, USA) in PBS without Ca^2+^ and Mg^2+^. Spectral fitting parameters for NADH and FAD were obtained by fitting emission spectra of corresponding reference solutions acquired through the same microspectroscopy setup. These were compiled into library files that could be applied in spectral decomposition of cellular autofluorescence spectra. A plot illustrating the result of said spectral decomposition is shown in Fig 1(inset).

From each autofluorescence spectrum, a redox ratio (RR) was computed as:

$$\begin{array}{c} \text{RR}=\frac{\left[\text{FAD}\right]}{\left[\text{FAD}]+[\text{NADH}\right]} \end{array}$$ (1)

The concentration of each cell-endogenous fluorophore (Fl) is related to its total fluorescence emission by:

$$\begin{array}{c} \left[\text{Fl}\right]=\frac{\int I_{Fl}d\lambda }{I_{0}\epsilon_{Fl}\phi_{Fl}L} \end{array}$$ (2)

where ∫*I*_*Fl*_*d*λ is the sum of intensities over the entire wavelength span of the fluorophore’s emission; *I*_0_ is the input excitation intensity; *ϵ*_*Fl*_ is the fluorophore’s extinction coefficient at the excitation wavelength; *ϕ*_*Fl*_ is the fluorophore’s quantum yield; and L is the path length of interaction between the input excitation and fluorophore. Substituting this into the RR and simplifying gives an optical variation of the RR:

$$\begin{array}{c} \text{RR}=\frac{\int I_{FAD}d\lambda }{\int I_{FAD}d\lambda +\int I_{NADH}d\lambda \times \frac{\epsilon_{FAD}\phi_{FAD}}{\epsilon_{NADH}\phi_{NADH}}} \end{array}$$ (3)

where $\frac{\epsilon_{FAD}\phi_{FAD}}{\epsilon_{NADH}\phi_{NADH}}$ is a constant that was experimentally determined.

To determine the constant, spectra of known mixtures of FAD and NADH were acquired in the microspectroscopy setup. For each known mixture, a RR was computed using Eq 1. A range of RR, from 0.017 to 0.580, were achieved by mixing different concentrations of NADH and FAD over the ranges of 0.9 to 14.4×10^−5^M and 0.6 to 50.0×10^−6^M respectively. For each acquired spectra, spectral decomposition would be performed so as to obtain ∫*I*_*FAD*_*d*λ and ∫*I*_*NADH*_*d*λ. The constant can then be solved for by simply substituting known values of RR, ∫*I*_*FAD*_*d*λ and ∫*I*_*NADH*_*d*λ into Eq 3. This was performed for 12 different RR values, with at least triplicate measurements for each.

### Bio-chemical assays

Two bio-chemical assays were conducted for the quantification of NADH and FAD using the NAD/NADH-Glo^™^Assay Kit (G9071, Promega Corporation, USA) and FAD Assay Kit (ab204710, Abcam, United Kingdom), correspondingly. For both assays, cells were seeded at concentrations of 1.5, 1.9, 2.3, 2.7, 3.2, 3.5×10^4^cells/ml in 6 separate 75cm^2^ cell culture flasks (Corning, USA) to achieve a range of confluencies between 20% and 90% after 1 day of incubation. Cells were prepared according to the respective assay kit’s protocols and all measurements were made using the multiplate reader. Fluorescence intensities were measured for emission at 587nm under excitation at 535nm for the FAD assay, while luminescence intensities were measured for the NADH assay. Each cell sample was measured concurrently with a set of standards so as to ascertain the total intracellular concentrations for both NADH and FAD. The intracellular concentrations per cell were subsequently determined for each measurement by accounting for the different extents of dilutions. This was conducted over three biological repeats per confluency.

### PrestoBlue viability assay

Cells were seeded at varying concentrations of 1.5, 2.0, 2.5, 3.0, 3.5×10^4^cells/ml in a 24-well culture plate (Corning, USA) to achieve a range of cell confluencies between 20% and 90% over a period of 4 days. Cell viability was assessed each day using a 10% PrestoBlue solution (Thermo Fisher Scientific, USA). This was done by adding the PrestoBlue solution to wells with cells that have reached the desired confluencies and subsequently incubating for 1h. 100*μ*l of media was then transferred to a black 96-well plate (Corning, USA) before fluorescence intensity measurements in a multiplate reader (Tecan Infinite M200 Pro, Thermo Fisher Scientific, USA). Measurements were made at 560nm excitation with emission collection at 590nm. This was conducted over three biological repeats, with at least three sets of data per reported confluency.

### Live-dead assay

A cell viability assay kit (LIVE/DEAD Viability/Cytotoxicity Kit, Invitrogen, USA) was used in the determination of live and dead cells. From the kit, a solution comprising 10*μ*l of red-fluorescent Ethidium Homodimer-1, 2.5*μ*l of green-fluorescence Calcein-AM and 5ml of PBS was prepared. Samples were then incubated with 500*μ*l of this solution at 37°C in the CO_2_ incubator for 20min. Samples were washed twice with PBS and 200*μ*l of live cell imaging solution was added. Stained cells were imaged using the same fluorescence microscope under 4× magnification. Live and dead cells were identified through the standard FITC and TRITC filter cubes, correspondingly. The images were processed using ImageJ and cell viability was calculated as (number live cells)/(number of live cells + number of dead cells)×100%. The live-dead study was conducted on samples following microspectroscopy. Negative control samples were not exposed to any form of illumination in the fluorescence microscope. >500 cells were imaged per sample.

### DNA damage assay

In DNA damage studies, cells were prepared and seeded within silicone wells on glass coverslips in the exact same manner as that for autofluorescence microspectroscopy. At 60% confluency, media within the wells were removed and replaced with 100*μ*l of live cell imaging solution (A14291DJ, Thermo Fisher Scientific, USA). Samples were then exposed to illumination with the same excitation used in autofluorescence microspectroscopy. This was performed for durations of 20s, 5min and 15min, together with a randomized scanning across the cell coverage area. Negative controls were set up in which samples were not exposed to any form of illumination in the fluorescence microscope. Samples were then washed twice with PBS and incubated with 1 ml of media at 37°C in the CO_2_ incubator for 6h. Samples were washed twice with PBS, fixed with 4% para-formaldehyde (28906, Thermo Fisher Scientific, USA) and permeabilized with 0.25% Triton-X (28313, Thermo Fisher Scientific, USA) in PBS. Samples were blocked with 5% BSA (A9418, Merck, USA) in 0.1% Triton-X in PBS and stained overnight with 1:250 Alexa Fluor488-conjugated anti-*γ*-H2AX (9719, Cell Signaling Technology, USA) diluted in 0.1% Triton-X in PBS. After antibody incubation, samples were washed with 0.1% Triton-X in PBS. Samples were stained for 5min with NucBlue Live ReadyProbes Reagent (R37605, Thermo Fisher Scientific, USA) and washed with PBS. Images were captured using an inverted fluorescence microscope and analyzed with CellProfiler (Broad Institute, USA). This was conducted over three biological repeats.

## Results

### Experimental constant in redox ratio calculations

The constant, $\frac{\epsilon_{FAD}\phi_{FAD}}{\epsilon_{NADH}\phi_{NADH}}$, in Eq 3 was determined to be 4.0±1.1 (S.D., N = 83). An average R-squared (R^2^) value of 0.996 was noted from the curve fitting achieved in spectral decompositions. This constant was used in all subsequent calculations of redox ratios (RR) from cellular autofluorescence spectra.

### Redox ratio at different cell confluencies

**Fig 2:**
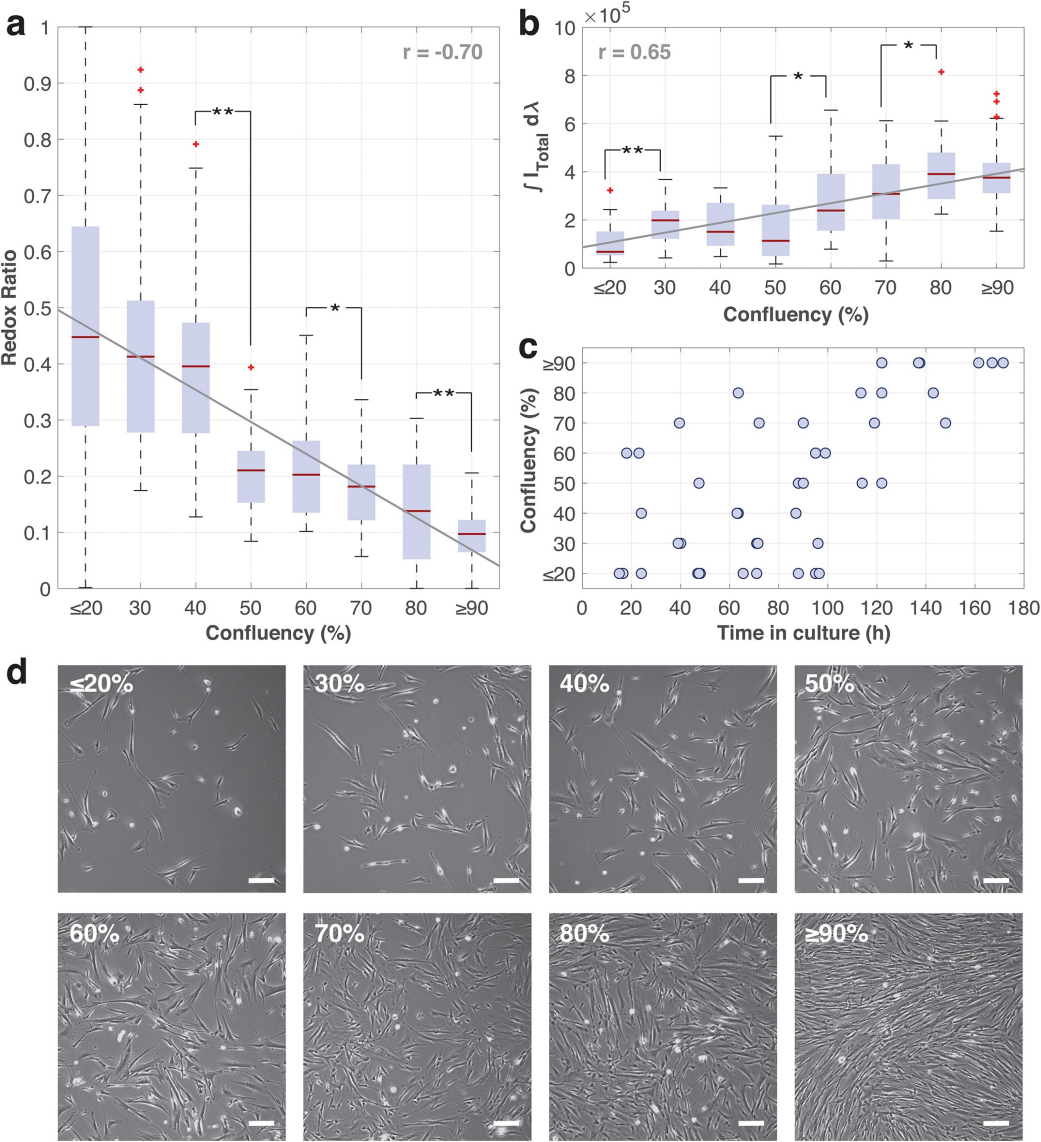
Measurements at different confluencies. Box and whisker plot of (a) redox ratios and (b) sum of total autofluorescence emission (from a ∼100*μm*^2^ area) for varying confluencies of fibroblasts cultured on glass coverslips. (c) Scatter plot of confluencies against time that fibroblasts spent in culture. (d) Corresponding phase contrast images of fibroblasts at different confluencies. In (a) and (b), each confluency interval comprises at least ten spectral measurements acquired from at least three technical repeats from the four biological repeats. Red horizontal lines within the box plots correspond to the median values. Blue shaded boxes indicate the interquartile range (IQR), while the black dotted whiskers demarcate the maximum and minimum values capped at 1.5×IQR. Red ‘+’s represent data points are outliers. Grey solid lines are the best linear fits with their corresponding Pearson correlation coefficients indicated by r. Statistical analysis: Data pairs marked with ‘*’ and ‘**’ are statistically significant based on a two-tailed Student’s t-test, where P<0.05 and P<0.001 respectively. Scale: White bars represent 200*μ*m.

RR was calculated for each of the acquired cellular autofluorescence spectra and organized in Fig 2a. The sum of total autofluorescence emissions (∫*I*_*Total*_*d*λ) was likewise collated in Fig 2b. Data was grouped according to the respective confluency of the cell samples they were collected from. Confluencies were determined through a side-by-side comparison of phase contrast images captured at 4x magnification as shown in Fig 2d. This was done at an accuracy of 10% between 30% and 80%. It was however difficult to discern between the very low confluency of ≤20% and the near-maximum confluency of ≥90%. Hence, data within these spans were further clustered.

In Fig 2a, we noted that the median RR ranges 0.39 to 0.45 for cell confluencies of ≤40%; 0.18 to 0.22 for cell confluencies between 50 and 70%; and 0.09 to 0.14 for cell confluencies of ≥80%. A general decrease in RR with higher cell confluency was observed. Statistical analyses comparing RR data of adjacent cell confluencies showed statistical significance between cell confluencies of 40% and 50%, 60% and 70% as well as 80% and ≥90%—P<0.001, P<0.05 and P<0.001 respectively, using a two-tailed Student’s t-test. Linear fitting of RR and confluency resulted in gradient of -0.0057 with a Pearson correlation coefficient of -0.70.

In Fig 2b, the median total autofluorescence emission was observed to generally increase with with higher levels of confluency from 0.7 to 3.9×10^5^. A dip in total autofluorescence emission was also noted at the cell confluency of 50%. Here, statistical analyses comparing total autofluorescence emission data of adjacent cell confluencies showed statistical significance between cell confluencies of ≤20% and 30%, 50% and 60% as well as 70% and 80%—P<0.001, P<0.05 and P<0.05 respectively, using a two-tailed Student’s t-test. Linear fitting of total autofluorescence emission (normalized to the highest value) and confluency resulted in gradient of 0.0050 with a Pearson correlation coefficient of 0.65.

### Autofluorescence images and redox ratio

**Fig 3:**
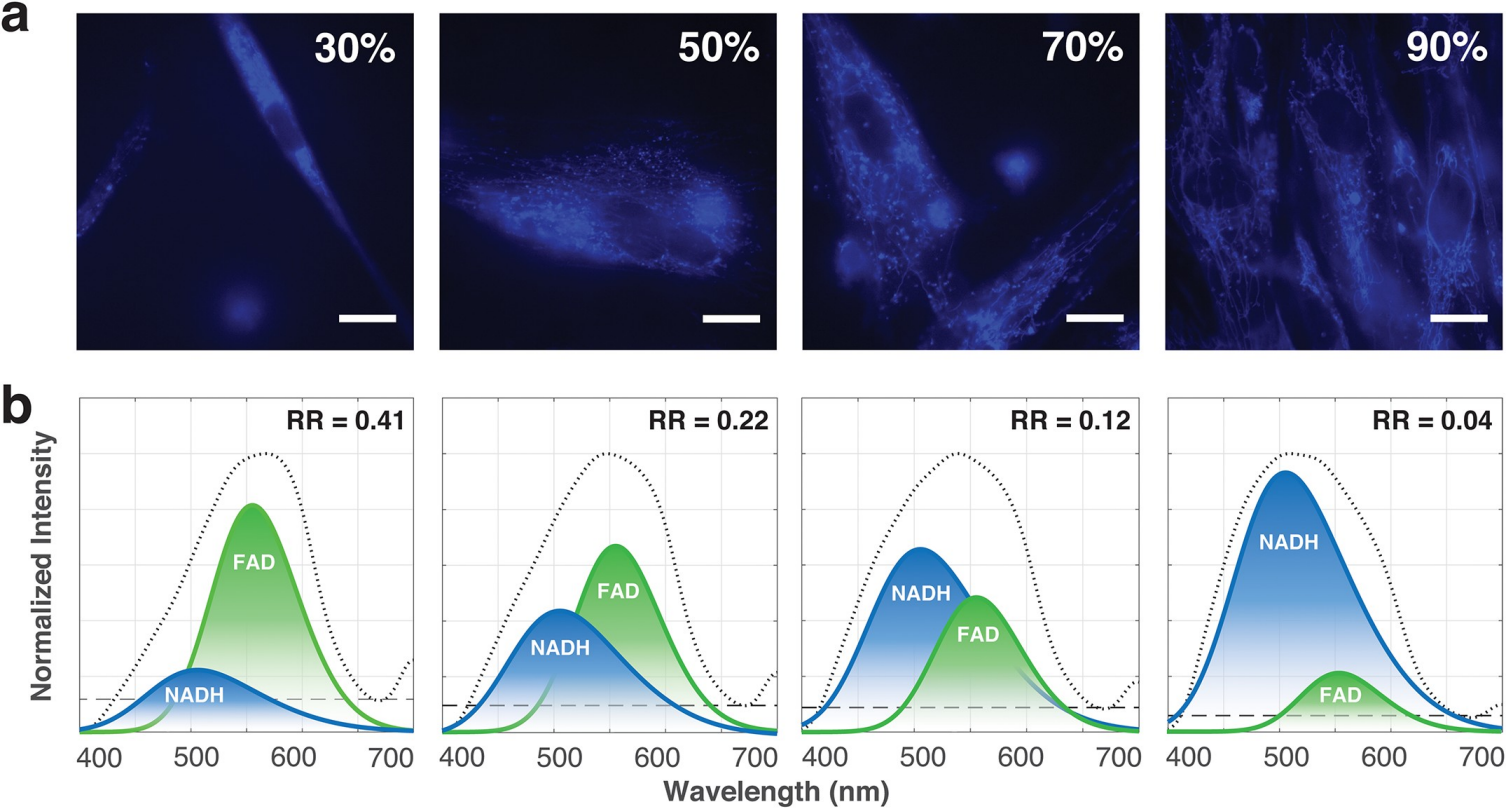
Changes in cellular autofluorescence at different confluencies. Representative cellular autofluorescence (a) images and (b) corresponding decomposed spectra at confluencies of 30%, 50%, 70% and 90%. In (b), black-dotted line represents the measured spectra. The blue and green curves indicate the constituent emissions by NADH and FAD respectively; while the black-dashed line is a constant component used in the fitting. Corresponding redox ratios (RR) are indicated for each decomposed autofluorescence spectrum. Scale: White bars represent 20*μ*m.

Corresponding changes in mitochondrial organisation were also noted from the autofluorescence images captured. Fig 3a depicts representative morphologies of cells and their mitochondria at different cell confluencies. We observed the transition of mitochrondrial organisation from being fragmented and distributed (at 30% confluency) to fragmented but concentrated around the nucleus (at 50% confluency) to interconnected and distributed (at 70% and 90% confluency).

### Validation with assays

**Fig 4:**
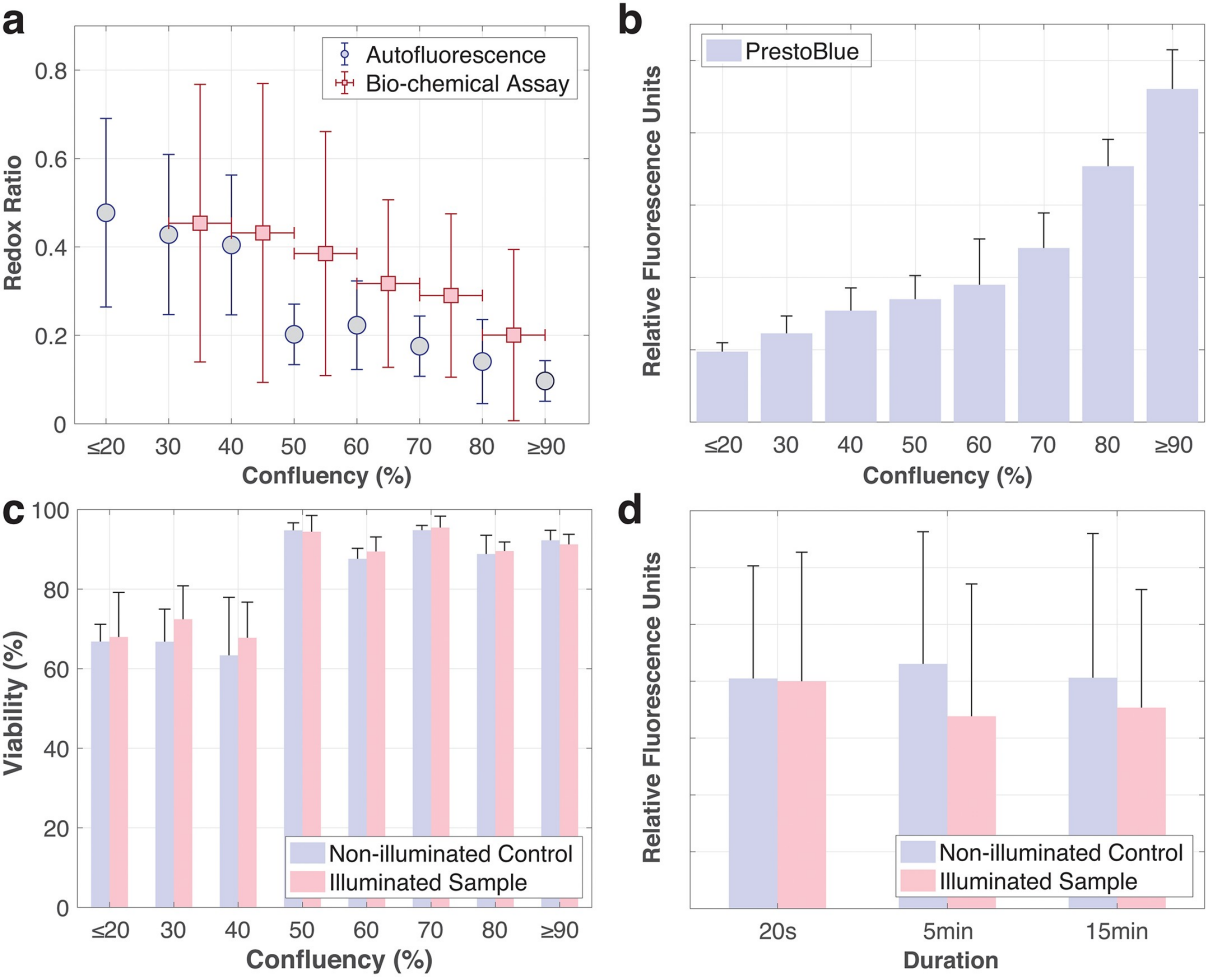
Assay data. (a) Redox ratios determined from bio-chemical assays (in red) and autofluorescence microspectroscopy (in blue) for varying confluencies. Vertical error bars represent standard deviation for N≥3. Horizontal error bars indicate confluency ranges due to variations in 75cm^2^ cell culture vessels. (b) PrestoBlue assay results at different confluencies. Error bars represent standard deviation for N = 3. (c) Viability assay results of cells following microspectroscopy (Illuminated sample) and corresponding controls (Non-illuminated control) for varying confluencies. Error bars represent standard deviation for five images taken for N = 1. Statistical analysis: One-tailed Student’s t-test between illuminated samples and their corresponding non-illuminated controls yielded no statistically significant pairs, where P>0.05. d) *γ*-H2AX assay results for cells following continuous durations (20s, 5min, 15min) of illumination (Illuminated sample) with corresponding controls (Non-illuminated control). Error bars represent standard deviation for N = 3. Statistical analysis: One-tailed Student’s t-test between illuminated samples and their corresponding non-illuminated controls yielded no statistically significant pairs, where P>0.05.

We further determined RR from bio-chemical assays, where the concentrations of NADH and FAD per cell were quantified bio-chemically at different confluencies as summarized in Table 1. These data are also overlaid with RR determined from autofluorescence spectroscopy in Fig 4a. It should be noted that confluencies were less specifically determined here because of variations across the larger 75cm^2^ cell culture flasks where these cells were cultured to their desired confluencies—in contrast to the smaller ∼1.9cm^2^ area in our silicone wells for microspectroscopy. Here, we observe RR to behave similarly to that obtained through autofluorescence spectroscopy.

**Table 1:** NADH and FAD concentration (per cell) computed from results of bio-chemical assays.

Confluency^†^ (%)	[NADH]/cell (×10^−5^M)	[FAD]/cell (×10^−5^M)	Redox Ratio
30–40	7.0±5.2	5.8±3.0	0.45±0.31
40–50	7.5±6.1	5.7±3.3	0.43±0.34
50–60	8.0±6.2	5.0±2.5	0.39±0.28
60–70	9.3±5.9	4.3±1.7	0.32±0.19
70–80	9.9±7.0	4.1±1.5	0.29±0.19
80–90	15±17	3.8±1.5	0.20±0.19

^†^Confluencies are reported in ranges due to variations across each 75cm^2^ cell culture vessel.

In addition, we conducted PrestoBlue viability assays to measure metabolic activity of cells at different confluencies. The measured fluorescence intensities corresponded were observed to increase with increasing cell confluencies as shown in Fig 4b. Cell confluencies were determined via bright-field microscopy over each 1.9cm^2^ area in the 24-well plate that they were cultured in.

Following microspectroscopy, we performed a live-dead assay to ascertain that the duration of exposure to illumination did not compromise cell viability. The percentage of live cells was determined for both the illuminated cell samples and corresponding controls. Cells used for control samples came from the same batch of culture and were prepared and exposed to the same treatments with the exception of any illumination from the fluorescence microscope. This study was conducted across the same range of cell confluencies as shown in Fig 4c. Statistical analysis conducted using a one-tailed Student’s t-test between the viabilities of the illuminated samples and their corresponding controls, revealed no statistical significance between each pair. However, statistical significance, via a two-tailed Student’s t-test, was observed between data of adjacent cell confluencies. Additionally, cell viabilities were observed to be ∼70% for lower levels of confluency (≤40%) and ∼90% for higher levels of confluency (≥50%).

To further determine damage induced by illumination, we conducted a study comparing levels of *γ*-H2AX, a marker for DNA double strand breaks [26], in cells prepared identically to our mircospectrocopy measurements with and without exposure to illumination. Cells were exposed to different durations of illumination at 20s, 5min and 15min. The shortest duration is representative of the shortest time required for 10 successive 2s measurements, while the longest duration is the maximum time required to complete the measurement of a sample. Results from the studied are shown in Fig 4d. Statistical analysis using a one-tailed Student’s t-test between illuminated samples and their corresponding controls revealed no statistical significance (P>0.05).

## Discussion

Autofluorescence spectra were signal processed and decomposed into the emission spectra of two cell-endogenous fluorophores—NADH and FAD. An excitation wavelength of 355nm was chosen because both NADH and FAD are capable of absorbing it, allowing both their fluorescence emissions to be collected from a single exictation. Spectral decomposition was based upon the method detailed by Croce *et al*. [10, 23]. In brief, we fitted the collected spectrum with two asymmetric Gaussian curves with peaks at ∼505nm and ∼555nm, corresponding to the emissions of reference samples of NADH and FAD measured in the same optical configuration. We note that in the work by Croce *et al*. multiple emission spectra (≥4) were used to achieve fits with R^2^ values of ∼0.99. In this work we simplified the fitting to just the two key cell-endogenous fluorophores, and made the assumption of negligible spectral differences (under our optical configuration) between free and bound NADH. In doing so, we noted R^2^ values of 0.95±0.01. Although less perfect fits were obtained, we find it sufficient in computing simple ratiometric relationships such as the redox ratio.

Results in Fig 2a show that RR generally decreases for higher levels of confluency. We attribute this observation to the different type of metabolic requirements necessary within cells as they switch from individual survival to concerted proliferation [27, 28]. During individual survival, oxidative metabolism dominates as glucose is consumed to generate biomass and produce energy—in the form of adenosine 5’-triphosphate (ATP). We expect this to happen for freshly seeded cells, as they adapt to the new environment and begin forming connections with the substrate and each other. Oxidative metabolism consumes NADH and generates FAD causing RR to increase; This has been reported to be a hallmark of differentiated cells [13, 29, 30]. Subsequently, as the cells enter a proliferative state they switch to anaerobic metabolism, where glucose is consumed and NADH is produced, with other metabolic precursors required for biosynthesis and cell replication are created in the same TCA cycle [31]. In contrast to oxidative metabolism, anaerobic metabolism increases the amount of NADH and decreases FAD, resulting in a lower RR. Lower RR has also been linked to cell proliferation [14] and higher anabolic activities [29].

Statistical analysis show that RR changes significantly when cell confluency increases from 40% to 50%, 60% to 70% and 80% to ≥90%. This transition in RR therefore can serve as a marker during cell manufacturing that can aid the operator in two ways: (i) allow objective prediction of a critical time for subculture, that is typically prescribed at a cell confluency of 70%; (ii) provide an early indicator for a go-no-go evaluation by determining the quality of cells based on growth rate, which can be calculated from the time required to reach a specific cell confluency. We further note that without spectral decomposition, the total autofluorescence emission (Fig 2b) possess a complementary set of significant changes when cell confluency increases from ≤20% to 30%, 50% to 60% and 70% to 80%. This could potentially be used in conjunction with RR to determine cell confluencies from cellular autofluorescence.

Comparison between linear fits in Fig 2a and 2b show a more sensitive response in RR than total autofluorescence emission—gradients of 0.0057 and 0.0050 respectively. In addition, we also note a stronger linear correlation between RR and cell confluency than total autofluorescence emission and cell confluency—Pearson correlation coefficients of -0.70 and 0.65 respectively. These indicate that RR is a better measure than the total autofluorescence emission. Although the sensitivity and linear correlation are only marginally higher for RR, it should be noted that RR is insensitive to intensity fluctuations as it is normalized and unitless. This implies that typical intensity variations as a result of optical misalignment or random artefacts are not going to affect the value of RR, making it a more a robust measure than the intensity-based measurement of total autofluorescence emission.

Variability was observed not only between different batches of biological repeats, but also within the batch and even amongst cells on the same coverslip. These variabilities are attributed to cellular heterogeneity where cells existed at different stages of the cell cycle, despite still collectively being part of a specific cell confluency. This is further illustrated by the spread of RR and total autofluorescence emission data in the boxplots of Fig 2a and 2b, correspondingly. The effect of cells coming from different batches was similarly presented by the clear lack of correlation between time in culture and cell confluencies shown in Fig 2c. Notably, these variabilities were more prominently presented by variations in RR in Fig 2a. We observe that the variation of RR is generally larger for lower levels of confluency (≤50%), with data for cell confluencies of ≤20% even spanning the entire range of 0 to 1. In that regard, it is recommended that RR data only be used for cell confluencies ≥30%. Nevertheless, it should be noted that such heterogeneity is to be expected in cell manufacturing as cell cycles are typically not synchronized prior to the expansion process.

Corresponding changes in mitochondrial organisation were also noted from the autofluorescence images captured. Fig 3a depicts representative morphologies of cells and their mitochondria at different cell confluencies. We observed the transition of mitochrondrial organisation from being fragmented and distributed (at 30% confluency) to fragmented but concentrated around the nucleus (at 50% confluency) to interconnected and distributed (at 70% and 90% confluency). Fragmented mitochondria exist in cells entering division [32] and in cells that are less metabolically active [33]. These correspond to the higher RRs observed at cell confluencies of ≤40%, and concurs with the reported inverse relationship between RR and metabolic activity [14]. Conversely, mitochondria become interconnected in a fused and branched organisation when cells are metabolically active [33]. This has also been related to health and bioenergetic efficiency [34] as well as used as an indicator of differentiation [15]. Although the autofluorescence images in Fig 3a are representative of their corresponding levels of confluency, we wish to highlight that the earlier mentioned variabilities definitely still exist.

Phases of cell cycle also contributes to the RR as redox has been reported to play several significant roles during the cell cycle leading to RR fluctuations. Maintenance of the redox state was observed to be important during G0/G1 to S phase transition so as to not affect redox-sensitive cell cycle regulatory proteins [35]; whereas an overall reduced environment is preferred during the G2/M phase so as to confer protective effects against oxidative damage of nuclear DNA during break down of the nuclear envelope in preparation for mitosis. These studies concur with our observation of a dip in RR for cell confluencies above 50%, coinciding with the switch into a proliferative state and having more cells going through mitosis. Similarly, in Fig 3, RR decreased from ∼0.4 to ∼0.2, with cells in the latter having binucleated phenotype, an indication of mitosis.

In our validation studies using standard assays, RR obtained from autofluorescence microspectroscopy and bio-chemical assays showed the same trend of a decreasing RR at higher cell confluencies, as depicted in Fig 4a. We noted a good agreement for low levels of confluency, while disparities existed at higher levels. These disparities could be attributed to higher heterogeneity in the 75cm^2^ flasks, where different cell confluencies could have existed at the point of the assay. This distribution would inevitably become more prominent at higher levels of confluency where multiple different clusters within the flasks were given time to expand at different rates into multiple different confluencies. This suggests that a pool of different FAD or NADH values was measured via the assays, in comparison to a dozen or so cells measured via autofluorescence microspectroscopy. Additionally, cells measured via the assay were handled differently, with steps like trypsinization and washing possibly contributing to the disparities. These also explain the observably larger spans in standard deviations of the bio-chemical assays. Here, we wish to also highlight that each assay-based measurement destructively consumes at least 1×10^6^ cells and takes at least 4h to run. On the contrary, each set of microspectroscopy-based measurement can be completed non-destructively in under 15min—inclusive of sample preparation, ≥10 spectral acquisitions and background measurements.

PrestoBlue assay results in Fig 4b show an inverse relationship to RR. The assay is a fluorescent-based assay that uses the reducing power of viable cells to quantitatively measure the proliferation of cells, where the measured fluorescence intensity is directly proportional to the number of metabolically active cells. With this assumption, a linear relationship would be expected between fluorescence intensity and cell confluency or numbers [36]. However, an exponential relationship was observed, suggesting that cell metabolic activity does not remain constant and instead increases at higher levels of confluency, also implying that cells become more reductive. This observation concurs with the inverse relationship expected between RR and metabolic activity [14]. In addition, we also observed that the initial cell confluency at time of seeding has negligible effect on cell metabolism measurements. We seeded different samples of cells at different starting confluencies and observed them to have similar metabolic activity (as measured by the PrestoBlue assay when they have reached the same levels of confluency. Similar to the bio-chemical assays, this assay takes a substantial amount of time to conduct (up to 1.5h) and although it is non-destructive, it still entails labelling.

From the live-dead assay results in Fig 4c, we conclude that the UV-illumination did not affect cell viability. On the other hand, we do note statistical significance between the cells at lower (≤40%) and higher (≥50%) levels of confluency. We attribute this to cells being less susceptible to death at higher levels of confluency when they have acclimatized, are healthier and more metabolically active. The greater extent of cell-cell contact at higher levels of confluency also enhances paracrine effects, which could further affect redox states of the cells. Secretion of factors or reactive oxygen species (ROS) molecules into the extracellular environment would affect nearby cells through direct uptake or the stimulation of redox pathways. These events would thus change redox states of the cells more drastically at higher levels of confluency where cells are closer in contact with each other. [37] Interestingly, this significant change from 40% to 50% in cell viability also matches the first significant change in RR (Fig 2a). This could be related to the transition of cells into a proliferative state from cell confluencies of 50%, following their acclimatization.

Additional study on DNA damage was conducted as the excitation wavelength used in our autofluorescence microspectroscopy measurements falls within the ultraviolet (UV) band. UV exposure is known to induce adverse effects on DNA through direct formation of lesions or indirect photochemical reactions. Results from our DNA damage study indicate no statistical significance between the illuminated samples and their corresponding non-illuminated controls. This could be attributed to the fact that the excitation wavelength (355nm) used falls in the UV-A band (315nm–400nm), which has been reported to have poor efficiency in inducing DNA damage [38]. These results further bolster the non-destructive nature of our measurements. Nevertheless, there is still the possibility that illumination could have other effects on cells such as photochemical degradation (especially in flavins [39]). This can however, be mitigated through the use of pulsed laser sources or more sensitive detectors, both of which reduces the effective exposure of cells to illumination.

Admittedly, the acquisition of cellular autofluorescence through polystyrene labware is challenging as thermoplastics emit a similar autofluorescence [40]. Although this will hinder the implementation of autofluorescence measurements in standard culture vessels, strategies such as multiphoton excitation at near infrared wavelengths offer spatial localization [41] that can avoid the undesired excitation of the polystyrene walls. Together with optical waveguide-based approaches, such localized excitations can be scaled to macroscopic areas to facilitate high throughput measurements [42]. Employing multiple excitations of different wavelengths could possibly also help mitigate problems with background by providing more data for referencing and correction. Alternatively, sterile closed-loop sampling of cells from the culture vessel could also circumvent issues pertaining to thermoplastic autofluorescence. In this case, cells will be continuously drawn into an external optical chamber for autofluorescence measurements, but its application would be limited to cells in suspension. Another cheaper and simpler option will be the use of glass-bottom culture dishes for sacrificial cell samples cultured along-side a main culture vessel. Probing the sacrificial samples will hence provide some representative measurements of cells in the main culture vessel. Regardless of the implementation approach, the autofluorescence microspectroscopy basis would still enable the advantages of rapid, non-destructive and label-free cell measurements.

## Conclusion

In this work, we used the label-free method of autofluorescence microspectroscopy to determine the redox ratio (RR) in cells at different cell confluencies. Autofluorescence spectra were acquired from cells through an inverted fluorescence microscope with simple upgrades. Through spectral decomposition of these spectra we were able to determine relative compositions of NADH and FAD in cells and consequently the relative extents of reduction and oxidation respectively—as represented by RR.

Autofluorescence results showed a trend of decreasing RR with increasing cell confluency, concurring with the trend observed through standard bio-chemical assays. We attributed this relationship between RR and cell confluency to several inter-related mechanisms: (i) change of metabolism process (oxidative metabolism at low confluencies to anaerobic metabolism at high confluencies), (ii) change of mitochondria organization (fragmented at low confluencies to interconnected at high confluencies), (iii) change of population of cells in G2/M state (more mitotic cells at high confluencies).

RR obtained from autofluorescence measurements were noted to change significantly across cell confluencies, offering a label-free marker for monitoring cellular redox during cell proliferation. The inverse relationship between RR and cell metabolism was further verified through bio-chemical and viability assays, allowing RR measurements to be a surrogate for cell metabolism. In comparison to current standards of using bio-chemical and viability assays, our microspectroscopy method was able to obtain the same results while reducing measurement times from hours to just minutes without affecting viability and DNA integrity of the cells.

In conclusion, we demonstrated a rapid, non-destructive and label-free method of measuring cell metabolism and redox state. We further established the relationship between cell metabolism and redox to proliferation, a critical measure of quality to be monitored in cell manufacturing. In addition, as redox state of cells are known to be important to several aspects of cell survival [43], monitoring redox can potentially also be used to determine oxidative stress levels in cells to track quality attributes like stemness [44] and senescence [45] in stem cell manufacturing. Lastly, the optical spectroscopy basis of the method allows it to be readily extended to meet the *in situ* and real-time monitoring requirements in cell therapy manufacturing.

## Supporting information

S1 Dataset Dataset (.xlsx) of results generated in this study. (XLSX)
